# Recanalization of a Severely Calcified Superior Mesenteric Artery in the Setting of Extensive Paravisceral Aortic Calcification Using Intravascular Lithotripsy: A Case Report and Focused Literature Review

**DOI:** 10.7759/cureus.101628

**Published:** 2026-01-15

**Authors:** Renato Abu Hana, Grit A Adler, Ruben G Ortiz Cordero, Oswaldo A Guevara Tirado, Vinicius Adami Vayego Fornazari

**Affiliations:** 1 Radiology, University of Florida College of Medicine, Jacksonville, USA

**Keywords:** chronic mesenteric ischemia, endovascular stenting, highly calcified stenosis, intravascular lithotripsy, shockwave intravascular lithotripsy, superior mesenteric artery recanalization

## Abstract

Calcified atherosclerotic plaques are a well-recognized cause of chronic mesenteric ischemia (CMI). We present a case where intravascular lithotripsy (IVL) was successfully used to treat a severely calcified and stenotic superior mesenteric artery (SMA). A 72-year-old male with diffuse atherosclerotic disease presented with acute-on-chronic abdominal pain and weight loss. Computed tomography angiography (CTA) demonstrated extensive calcified aortic atherosclerotic disease extending to the major branches, particularly the SMA, causing severe stenosis. Plain old balloon angioplasty (POBA) followed by Shockwave™ IVL enabled the placement of a stent graft, resulting in successful recanalization. Following the procedure, the patient reported significant improvement in abdominal pain. IVL provides a safe and effective method for managing severely calcified mesenteric lesions, particularly when standard angioplasty alone is insufficient. This case contributes meaningfully to the limited body of evidence supporting SMA-IVL and underscores its value in complex paravisceral arterial disease.

## Introduction

Chronic mesenteric ischemia (CMI) is an uncommon and frequently underdiagnosed vascular disorder that typically affects older individuals with diffuse atherosclerotic disease. Symptoms arise when blood flow through the mesenteric arteries is reduced by approximately 60-75%, leading to postprandial abdominal pain, weight loss, and decreased appetite [1-6]. One population-based cohort study by Hansen et al. estimated the prevalence of significant mesenteric artery stenosis to be approximately 17.5% in adults older than 65 years, with symptoms depending on available collateral circulation [7].

Definitive management for symptomatic patients aims to restore adequate mesenteric blood flow and includes either open surgical revascularization or an endovascular approach, such as percutaneous transluminal angioplasty (PTA) with or without stent placement [1-3,5-6]. However, in cases of severe arterial calcification, vessel compliance is reduced, making adequate vessel expansion and stent delivery technically challenging and potentially predisposing to stent underexpansion.

Intravascular lithotripsy (IVL) has emerged as a novel adjunctive therapy to address this limitation. The Shockwave™ IVL system uses emitters mounted on a specialized balloon catheter to deliver pulsed acoustic pressure waves that propagate through the vessel wall, selectively fracturing intimal and medial calcium while sparing surrounding soft tissues. This controlled calcium modification increases arterial compliance, allowing effective low-pressure balloon expansion and safer stent deployment [2-5].

IVL has demonstrated safety and efficacy in the treatment of calcified coronary, renal, and peripheral arterial disease [2,4]. However, literature documenting its application in the mesenteric circulation is limited. We present a case of a high-risk patient with mesenteric ischemia and a severely calcified proximal superior mesenteric artery (SMA) stenosis that was successfully treated with IVL and stenting, highlighting a rarely reported endovascular approach for mesenteric revascularization.

## Case presentation

**Figure 1:**
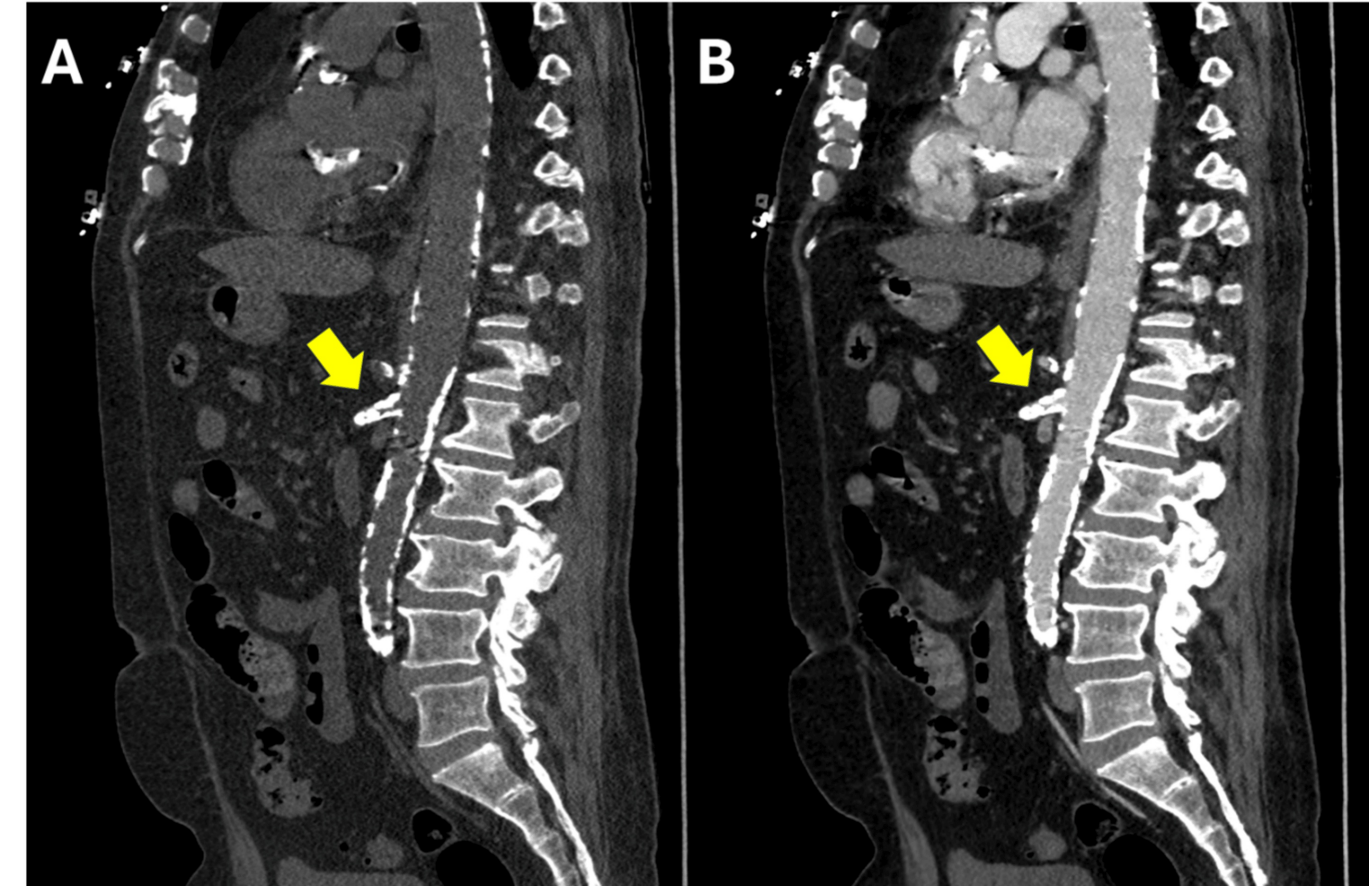
Sagittal (A) pre- and (B) post-contrast CT angiogram of the abdomen and pelvis demonstrating extensive calcification of the descending aorta and superior mesenteric artery (arrows).

A 72-year-old male with a past medical history significant for obesity, hypertension, dyslipidemia, 43-pound weight loss in the last six months, loss of appetite, and extensive atherosclerotic disease developed intermittent, diffuse, postprandial abdominal pain during an unrelated admission. The pain progressively worsened over a span of approximately four days, raising concerns for acute-on-CMI. Computed tomography angiography (CTA) of the abdomen and pelvis demonstrated extensive calcified atherosclerotic disease, resulting in severe multifocal stenosis, including the celiac artery and the SMA (Figure 1).

Interventional radiology was consulted to perform a mesenteric angiogram and possible SMA recanalization. Appropriate informed consent was obtained from the patient. Pre-intervention laboratory results were remarkable for leukocytosis (14.15×10³/µL) and anemia (7.5 g/dL).

**Figure 2:**
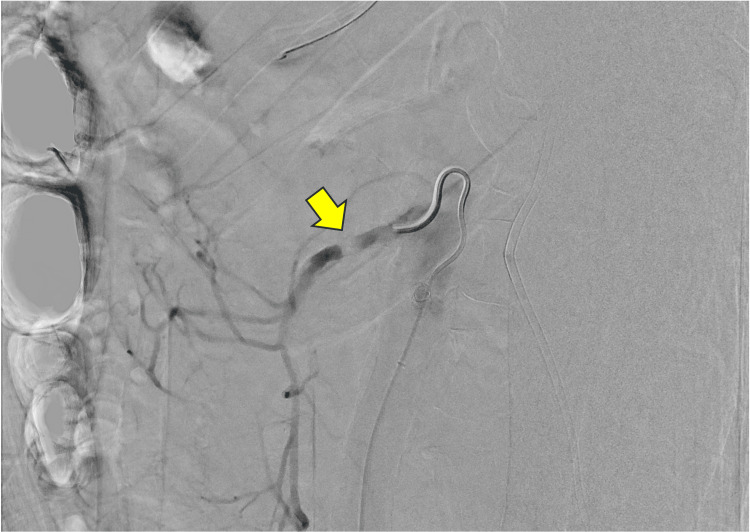
Pre-intervention angiogram of the superior mesenteric artery demonstrating extensive calcification and severe stenosis (arrow).

**Figure 3:**
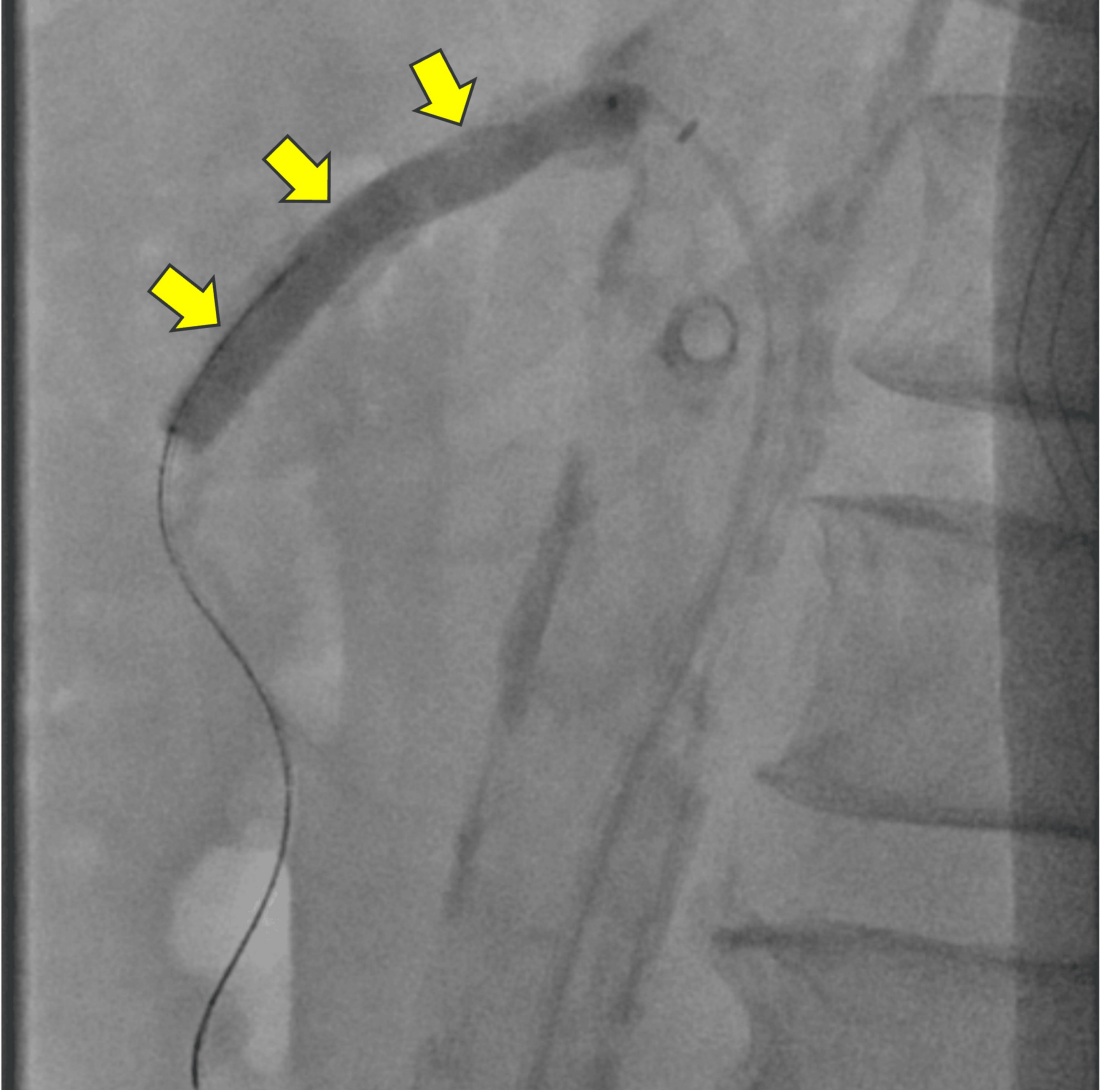
Angiogram demonstrating the Shockwave™ intravascular lithotripsy balloon (arrows) being placed across the proximal superior mesenteric artery.

A decision was made to use the right common femoral artery approach, which was accessed using a micropuncture needle, allowing the placement of a 6Fr Terumo Destination® sheath, 45 cm. Utilizing a 5F C2 catheter and a Terumo 0.035" Glidewire Advantage®, the SMA was selectively catheterized under fluoroscopic guidance. A digital subtraction angiogram of the SMA demonstrated high-grade calcified stenosis of the proximal portion of the SMA with approximately 90% luminal narrowing (Figure 2). Under fluoroscopic guidance, the narrowing within the proximal SMA was successfully crossed, and an angiogram was performed, confirming the true lumen position of the catheter. The remaining mesenteric branches arising from the SMA were widely patent. The 0.035" wire was replaced by an Asahi Astato® Xs 0.014" wire. Given the severe stenosis of the SMA, the Shockwave™ IVL balloon was not able to cross the lesion, and predilation was achieved with a 3 x 50 mm plain balloon. The balloon was removed, and a 6 x 60 mm Shockwave balloon was advanced into the proximal SMA (Figure 3). Three rounds of shockwave lithotripsy with a total of 120 cycles were performed.

**Figure 4:**
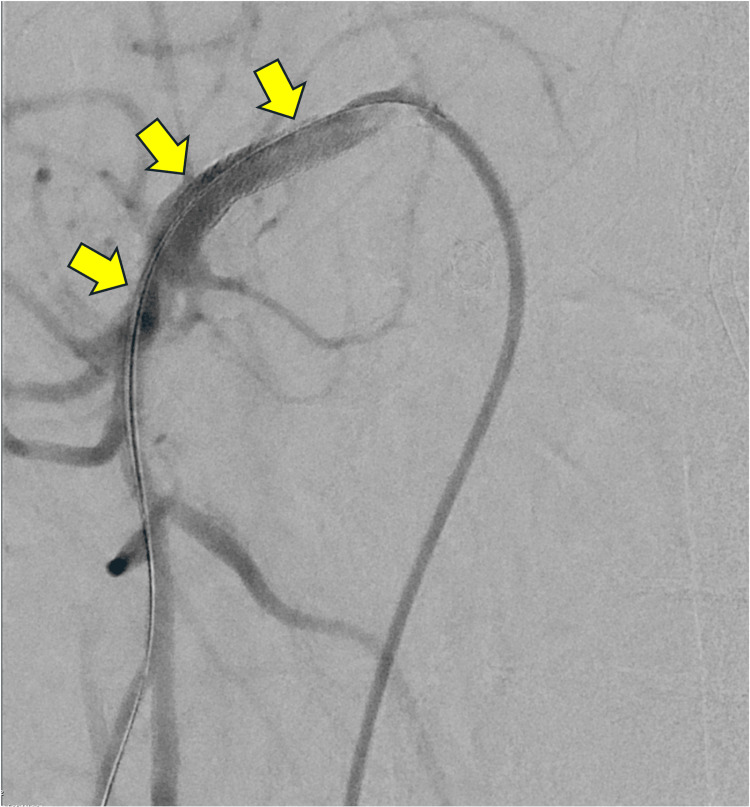
Final angiogram (zoomed-in view) after IVL and stenting demonstrating adequate flow through the superior mesenteric artery without evidence of significant residual stenosis; arrows indicate the treated segment. IVL, intravascular lithotripsy

Finally, an Abbott Omnilink Elite® vascular balloon-expandable uncovered stent measuring 6 x 39 mm was successfully deployed within the proximal SMA. The final angiogram demonstrated brisk flow through the SMA without evidence of significant residual stenosis (Figure 4). An Angio-Seal closure device was utilized for proper hemostasis of the access. A total of 80 mL of Omnipaque 300 was used. Ultimately, the SMA recanalization with balloon angioplasty, IVL, and stent placement was successful.

The following day, the patient expressed significant improvement in abdominal pain. The patient was to start aspirin and clopidogrel for three months, followed by lifetime aspirin use. Unfortunately, due to other comorbidities, including a recent stroke, the patient's clinical condition declined, and he expired prior to the next follow-up.

## Discussion

When left untreated, symptomatic CMI has a five-year mortality rate of almost 100%, emphasizing the need for prompt management [8]. Typical treatment options for CMI include open surgical revascularization, PTA, and stent placement, most commonly involving the SMA to restore adequate mesenteric perfusion and relieve ischemic symptoms [1]. Current evidence suggests that symptomatic CMI is best managed using endovascular techniques, such as IVL, which are associated with fewer perioperative complications [8].

Heavily calcified stenotic lesions of the SMA represent one of the most challenging scenarios in endovascular mesenteric revascularization. Traditional balloon angioplasty often fails due to inadequate vessel compliance, and high-pressure inflations carry risks of dissection or rupture.

IVL employs pulsatile energy to generate localized electrical discharges that vaporize the surrounding fluid, producing acoustic pressure waves that fracture calcified deposits within the arterial media while preserving the integrity of the intima [2-4]. This technique allows circumferential plaque modification with minimal vascular trauma and a low risk of distal embolization [2-4]. Rishad et al. demonstrated that IVL use in coronary interventions was associated with reduced procedural costs and decreased utilization of adjunctive devices such as balloons, guidewires, and stents [9].

IVL has been successfully applied in coronary, renal, iliac, and femoral arteries [3], and multiple recent reports have also described its effective use in the treatment of calcified lesions involving the SMA, underscoring its expanding role in mesenteric revascularization [2]. The safety and efficacy of IVL have been demonstrated in both coronary and peripheral applications. The DISRUPT PAD II and PAD III studies reported favorable outcomes, with primary patency rates of approximately 70% at 12 months and no significant complications such as distal embolization or thrombosis [3,10].

Compared with previously reported SMA IVL cases, including those by Balboa Arregui et al., Khan et al., and Spath et al., the present case uniquely demonstrates IVL feasibility in the setting of extensive paravisceral aortic calcification [3,4]. The need for predilation prior to Shockwave balloon deliverability, the choice of 120 cycles, and the subsequent successful deployment of a balloon-expandable stent highlight practical considerations for managing such complex lesions.

This case reinforces IVL as a valuable adjunct in mesenteric revascularization and supports further investigation into long-term patency, device safety, and the development of standardized protocols specific to visceral arteries. Additionally, our case expands upon existing literature by demonstrating successful IVL-assisted stent placement in the setting of extensive paravisceral calcification. However, it is important to note that a key limitation in our case was the absence of standardized clinical and imaging follow-up to confirm long-term patency and durability due to the patient's clinical decline and death.

## Conclusions

IVL may be considered when managing severely calcified mesenteric lesions, particularly when standard angioplasty alone is insufficient. By facilitating stent placement in heavily calcified vessels, IVL-assisted SMA stenting represents a promising potential treatment approach for patients with symptomatic mesenteric ischemia and extensive calcifications. This case contributes meaningfully to the limited body of evidence supporting SMA-IVL and underscores its value in complex paravisceral arterial disease. However, long-term patency and durability could not be assessed in our case. Therefore, continued clinical follow-up and additional studies are needed to better characterize long-term outcomes.
